# Association between sleep quality and nocturnal erection monitor by RigiScan in erectile dysfunction patients: a prospective study using fitbit charge 2

**DOI:** 10.1186/s12610-023-00206-x

**Published:** 2023-11-27

**Authors:** Yuyang Zhang, Wei Zhang, Xingliang Feng, Guodong Liu, Xu Wu, Hui Jiang, Xiansheng Zhang

**Affiliations:** 1 Department of Urology, the First Affiliated Hospital of Anhui Medical University, Anhui Province, China; 2 Institute of Urology, the First Affiliated Hospital of Anhui Medical University, Anhui Province, China; 3 Anhui Province Key Laboratory of Genitourinary Diseases, Anhui Medical University, Anhui Province, China; 4 Department of Urology, The Third Affiliated Hospital of Soochow University, Changzhou, Jiangsu China; 5 Department of Urology, The First People’s Hospital of Changzhou, Changzhou, Jiangsu China; 6 Department of Urology, Peking University First Hospital Institute of Urology, Peking University Andrology Center, Beijing, China

**Keywords:** Erectile dysfunction, Sleep quality, Nocturnal erection monitor, RigiScan, Fitbit Charge 2™

## Abstract

**Background:**

Few studies were conducted to explore the association between sleep quality and nocturnal erection. Here, we intended to explore the association between sleep quality and nocturnal erection monitor when conducting nocturnal erection monitor. All erectile dysfunction (ED) patients underwent sleep monitors using Fitbit Charge 2™ (Fitbit Inc.) and nocturnal penile tumescence and rigidity (NPTR) monitors using RigiScan® (GOTOP medical, Inc., USA) for two nights. Subsequently, the patients were divided into two groups: Group A included patients who experienced effective erections only on the second night, while Group B included patients who had effective erections on both nights. To explore the associations between NPTR parameters and sleep parameters, a comparative analysis was performed between Group A and Group B for both nights.

**Results:**

Finally, our study included 103 participants, with 47 patients in Group A and 56 patients in Group B. Notably, the Group A patients showed significant improvements in NPTR parameters on the second night compared to the first night. Conversely, the NPTR parameters on Group B of the second night did not demonstrate a superior outcome when compared to the second night of Group A. Interestingly, it was found that only the disparities in sleep parameters accounted for the variation in NPTR parameters between the two groups on the first night. After correlation and ROC analysis, we identified the rapid eye movement (REM) sleep time and wake after sleep onset (WASO) time monitoring by the Fitbit Charge 2 as the primary parameters for predicting abnormal NPTR results in the first night.

**Conclusions:**

Therefore, our study strongly suggests a close association between sleep parameters and NPTR parameters. It emphasizes the importance of incorporating sleep monitoring alongside nocturnal erection monitoring to enhance the reliability of the NPTR results.

## Résumé

**Contexte:**

Peu d’études ont été menées pour explorer l’association entre la qualité du sommeil et l’érection nocturne. Dans cette étude, notre but était d’explorer l’association entre la qualité du sommeil et le moniteur d’érection nocturne lors de l’utilisation d’un moniteur d’érection nocturne. Tous les patients atteints de dysfonction érectile (DE) ont été soumis à des moniteurs de sommeil utilisant Fitbit Charge 2™ (Fitbit Inc.) et à des moniteurs de tumescence et de rigidité péniennes nocturnes (NPTR) utilisant RigiScan® (GOTOP medical, Inc., États-Unis) pendant deux nuits. Par la suite, les patients ont été divisés en deux groupes: le groupe A comprenait les patients qui n’avaient eu des érections efficaces que la seconde nuit, tandis que le groupe B comprenait les patients qui avaient des érections efficaces à chacune des deux nuits. Pour explorer les associations entre les paramètres du NPTR et les paramètres du sommeil, une analyse comparative a été effectuée entre le groupe A et le groupe B pour les deux nuits.

**Résultats:**

Au final, notre étude a inclus 103 participants, dont 47 patients dans le groupe A et 56 patients dans le groupe B. Les patients du groupe A ont montré des améliorations significatives des paramètres de NPTR la seconde nuit par rapport à la première nuit. À l’inverse, les paramètres de NPTR de la seconde nuit pour le groupe B n’ont pas donné de résultats supérieurs à ceux de la seconde nuit du groupe A. Fait intéressant, il a été constaté que seules les disparités dans les paramètres de sommeil expliquaient la variation des paramètres de NPTR entre les deux groupes lors de la première nuit. Après corrélation et analyse ROC, nous avons identifié le temps de sommeil paradoxal et la surveillance du temps de réveil après l’endormissement monitorés par le Fitbit Charge 2 comme les principaux paramètres de prédiction des résultats de NPTR anormaux au cours de la première nuit.

**Conclusions:**

Par conséquent, notre étude suggère fortement une association étroite entre les paramètres de sommeil et les paramètres de NPTR. Elle souligne l’importance d’intégrer la surveillance du sommeil parallèlement à la surveillance de l’érection nocturne pour améliorer la fiabilité des résultats de NPTR.

**Mots-clés:**

Dysfonction érectile, Qualité du Sommeil, Moniteur d’Erection nocturne, RigiScan, Fitbit Charge 2™

## Introduction

Nocturnal penile tumescence (NPT), formerly known as sleep related erections (SREs), is a natural and spontaneous physiological phenomenon observed in healthy men during nighttime sleep across their lifespan [1]. While the precise mechanisms behind NPT remain unclear, it is widely recognized that the NPT is closely linked to the rapid eye movement (REM) sleep [2], and approximate 80% of NPT episodes occur during REM sleep following a cyclical pattern that repeats approximately every 80 min and lasts 20 min each time [3]. It has been presumed that the presence of normal NPT relies on the integrity of local penile tissues, as well as neural and vascular pathways responsible for sexually stimulated erections [4]. Therefore, normal NPT has been utilized as a means to differentiate the psychogenic erectile dysfunction (pED) from organic erectile dysfunction (ED), as it typically occurs during sleep without the absence of anxiety, stress, apprehension, and other psychological factors [5]. Based on the theory, various objective tools for assessing erection have been developed, including mercury-filled strain gauge recorder [6], the stamp test [7], the angle of erection in the standing position test [8], the axial penile buckling test [9], and the nocturnal penile tumescence and rigidity (NPTR) test [10]. Nowadays, the RigiScan [10] has emerged as the standard tool recommended by both the European Association of Urology (EAU) [11] and American Urological Association (AUA) guidelines [12] for assessing the circumferential change and the rigidity of NPT.

Nevertheless, it should be noted that not all NPTR results accurately reflected the actual condition of patients with ED. During the monitoring of nocturnal erections using the RigiScan® device (GOTOP medical, Inc., USA), a phenomenon, known as “first-night effect” has been observed [13]. This implies that certain patients may exhibit abnormal NPTR results during their initial measurement in a laboratory setting, but these results may be reversed when conducting consecutive nightly measurements [13]. The researchers concluded that the abnormal NPTR results observed during the initial measurement were attributed to reductions in sleep quality, including decreased REM sleep and intermittent wake times [14, 15]. And the reduced sleep quality was most likely caused by the discomfort and movement restrictions of the device, potential psychological effects associated with medical scrutiny, and alterations in the sleep environment [13]. In essence, the validity of abnormal NPTR results becomes questionable when sleep quality is compromised. However, there have been relatively few studies to substantiate this prevailing notion. The primary obstacle in conducting such studies has been the absence of dependable and objective sleep monitoring methods during concurrent NPT testing.

Polysomnography (PSG), is widely recognized as the gold standard for objective sleep assessment. And it could continuously record the electroencephalographic (EEG), electromyographic (EMG), and electrooculographic (EOG) activity using surface electrodes, to virtually score wake and sleep stages (N1, N2, N3, REM sleep) [16]. While PSG is highly accurate in assessing sleep, it is important to acknowledge that the sleep quality may be inevitably affected by the unfamiliar setting and the intensive monitoring process involving multiple sensors [17]. Furthermore, successful implementation of PSG requires professional sleep technicians, a dedicated PSG acquisition system, and technical specialists. So, it’s impractical and unsuitable to assess patients’ sleep utilizing PSG during nocturnal erection monitor. With the rapid development of the internet, intelligent hardware, and big data, the wearable technology has made significant progress and found applications in various fields, especially in the realm of healthcare, such as sleep and safety monitoring, exercise and fitness tracking. [18]. Contrary to complicated nature of PSG, wrist actigraphy offers a more suitable and practical method to record patients’ sleep. Among various sleep-trackers available in the consumer market, the Fitbit Charge 2™ stand out as an intelligent wearable device that employs a multisensory approach to accurately detect sleep–wake states [19]. The portable device demonstrates comparable accuracy to PSG in detecting sleep–wake stages and sleep stage composition, mainly in wake stage and REM sleep stage [19–22]. Furthermore, this device's lightweight and user-friendly design ensure that the sleep quality is not compromised during its usage [20]. Excitingly, the development of the wearable device overcome our barrier to detect the sleep quality without any adverse effects on sleep itself during nocturnal erection monitor [23].

In 2022, the Fitbit Charge device was firstly used by our group to compare the sleep parameters between ED patients and healthy controls [24]. We elucidated that abnormal sleep quality monitoring by the Fitbit device may be a risk factor for ED. Subsequently, we enrolled health individuals to explore the relationship between ﻿REM Sleep and SREs by Fitbit™ and RigiScan®. We verified that most REMs occurred during the REM sleep, which has been validated by Mann et al. combining PSG with RigiScan® [25]. Based on these findings, we intend to furtherly explore the association between sleep quality monitoring using Fitbit Charge 2™ and nocturnal erection monitoring using RigiScan® in patients with ED. The first purpose of our study is to confirm that the presence of the first-night effect observed during NPTR test is attributable to abnormal sleep quality when conducting the NPTR examination. The second objective is to identify sleep parameters of Fitbit monitor that can effectively predict abnormal NPTR results during NPTR examination.

## Materials and methods

This prospective study was conducted in the Department of Andrology and Urology of the First Affiliated Hospital of Anhui Medical University from June 2021 to August 2022, and the study protocol was approved by the Institutional Ethics Committee of our hospital in advance (Quick-PJ-2022–07-61). All the participants in the study provided informed consent before participating the study, which clearly outline the associated risks and benefits. It is important to note that participation in the study was completely voluntary.

### Study population and design

Men diagnosed with ED and aged 18 years or older were invited to participate in the study. The inclusion criteria for the study are as follows: (1) diagnosed with ED as the International Index of Erectile Function-5 (IIEF-5) ≤ 21; (2) aged older than 18 with a history of regular sexual intercourse and a stable heterosexual partner; (3) having enough time to finish two consecutive night measurements. Patients would be excluded from our study if they met the following exclusion criteria: (1) history of diabetes mellitus, hypertension, hyperlipidemia, and other cardiovascular disease; (2) history of obstructive sleep apnea, as well as other sleep disorders; (3) history of spinal cord injury; (4) intake of drugs affecting the erectile function, as erotogenic drugs, and antipsychotic drugs; (5) history of Hypogonadism.

Firstly, a comprehensive medical and sexual history would be collected by an andrologist, which included age, height, weight, and personal history of smoking, alcohol use, and regular exercise. Then, several questionnaires were administrated to preliminarily assess the erectile function, anxiety and depression, and sleep quality. The IIEF-5 questionnaire was used to assess the erectile function of participants, with a diagnostic threshold of 21 points or lower [26]. The General Anxiety Disorder‐7 (GAD‐7) was utilized to assess level of anxiety with scores below 5 generally considered within the normal range, and the Patient Health Questionnaire‐9 (PHQ‐9) questionnaire was used to measure the severity of depression system with scores below 5 also generally considered within the normal range [27]. Both questionnaires have been formally validated against diagnostic clinical interviews to determine the sensitivity and specificity [28, 29]. And the Insomnia Severity Index (ISI) was employed to assess sleep quality of the participants [30]. The Obstructive Sleep Apnea Screening Questionnaire was used to identify and exclude patients with obstructive sleep apnea syndrome. And those who scored ≥ 3 on the questionnaire were diagnosed as having obstructive sleep apnea and were excluded from the study [31].

After the medical inquiries and physical examinations, blood samples from all participants were collected from the antecubital vein in the morning period between 6:00 to 8:00, after an eight-hour fasting period. All measurements were performed within 1 h of venipuncture, including fasting blood glucose (FBG), total cholesterol (TC), triglycerides (TG), and total testosterone (TT). All patients underwent the following sleep monitors and NPTR monitors for two consecutive nights in the sleep unit of our department, which was specifically designed to perform various andrology tests for patients with sexual dysfunction.

### NPTR and sleep measurements

The NPTR monitoring was conducted using the RigiScan® device (GOTOP medical, Inc., USA), while sleep monitoring was performed using the Fitbit Charge 2TM device (Fitbit Inc., USA). On the days of examination, participants were instructed to abstain from any activities that could potentially interfere with sleep. These activities included napping, smoking, drinking tea, consuming caffeine or alcohol, and taking hypnotic medications. Additionally, participants were advised to empty their bowels and bladder before sleep to minimize the chances of defecation or experiencing nocturia during the monitoring period.

The RigiScan® device was secured to the patient’s inner thigh, with two self-calibrating loops attaching to the penis, one loop at the tip, and the other at the base. All measurements commenced at 22:00 PM and concluded at 7:00 AM the following day. The ‘RigiScan plus version 4.0’ software was used for the analysis of the NPTR results. The NPTR results that were collected and formally analyzed included the following parameters: total test time (TTT), effective erectile events (EEE), total erection time (TET), the duration of erectile episodes with rigidity ≥ 60% at both sites (Tip D60% and Base D60%), and the average event rigidity at both sites (Tip AER and Baes AER). According the guidelines regarding ED, an effective erectile event was defined as an erectile episode with penile tip rigidity ≥ 60% and a duration lasting longer than 10 min [32]. In this study, a patient was classified as having normal erectile function if at least one effective erectile event was recorded during the measurement period. However, if the total test time recorded by RigiScan® was less than 6 h, those patients would be excluded from the study.

After the correct adjustment of RigiScan, the patients simultaneously wore the Fitbit Charge 2 wristband. Fitbit Charge 2 is the wearable fitness tracker wristband capable of monitoring sleep [20]. The device could automatically connect to a mobile phone via Bluetooth, and transfer the data to the phone via a dedicated APP, named “Fitbit”. Every account, created via e-mail, could connect a Fitbit device. And the data of the corresponding Fitbit could be stored not only on the mobile platform, but also on the internet. The device was positioned on the patient’s non-dominant wrist, approximately one inch above the wrist bone. The device needs to be contacted with the skin properly, without movement or excessive pressure. The device screen was locked by our technician via password to prevent unintended activation of the screen's backlight. The data collected by the Fitbit device included total sleep time (TST), sleep onset latency (SOL), wake after sleep onset (WASO), time in light sleep (N1 + N2), time in deep sleep (N3), and time in REM. According to the guideline of American Academy of Sleep Medicine, sleep consist of various stages, namely stage N1, N2, N3, and REM sleep based on the ﻿electroencephalography examinations [33]. The N1 stage is defined as light sleep stage characterized by drowsiness and relaxation. The N2 stage is a deeper stage where the body prepares for deep sleep. The N3 stage, also known as slow-wave sleep, is the stage of deep and restorative sleep. The REM stage is a stage of sleep associated with vivid dreams and increased brain activity, which also characterized by rapid eye movements, irregular breathing, and muscle paralysis [34].

### Statistical analysis

Based on the results of NPTR examinations, all included patients were categorized into four types: type one with patients presenting effective erectile events only on first night; type two with patients presenting effective erectile events only on second night; type three with patients presenting effective erectile events on both night; and type four with patients presenting no effective erectile events on both nights. Patients without any effective erection during either night or with effective erectile events only on the first night, were excluded from our analysis. Then, the remaining patients were divided into two groups for further analysis. Patients who exhibited at least one effective nocturnal erection on the second night but did not have any effective nocturnal erections on the first night were assigned to Group A. Patients who had effective nocturnal erections on both nights were assigned to Group B. We conducted a comparative analysis of all NPTR parameters and sleep parameters between the first and second nights within each group. Additionally, we compared all NPTR parameters and sleep parameters between Group A and Group B for both nights.

Baseline characteristics of continuous variables were presented as mean ± standard derivation (SD), while categorical variables were presented as frequencies and percentages. For comparisons between groups, the independent sample t tests were employed for continuous variables that exhibited a normal distribution, as confirmed by the Kolmogorov–Smirnov test. And the Pearson chi-square tests were used to compare the categorical variables between groups. For comparisons within the same group between first night and second night, the paired sample t tests were employed, surely based on the assumption of sample normality. Correlations between the sleep parameters and NPTR parameters were evaluated by Spearman’s and Pearson’s method whenever appropriate. The receiver operating characteristic (ROC) analyses were conducted to assess the predictive ability of sleep parameters to predict abnormal NPTR results in ED patients. *P* < 0.05 was considered statistically significant.

## Results

**Fig. 1:**
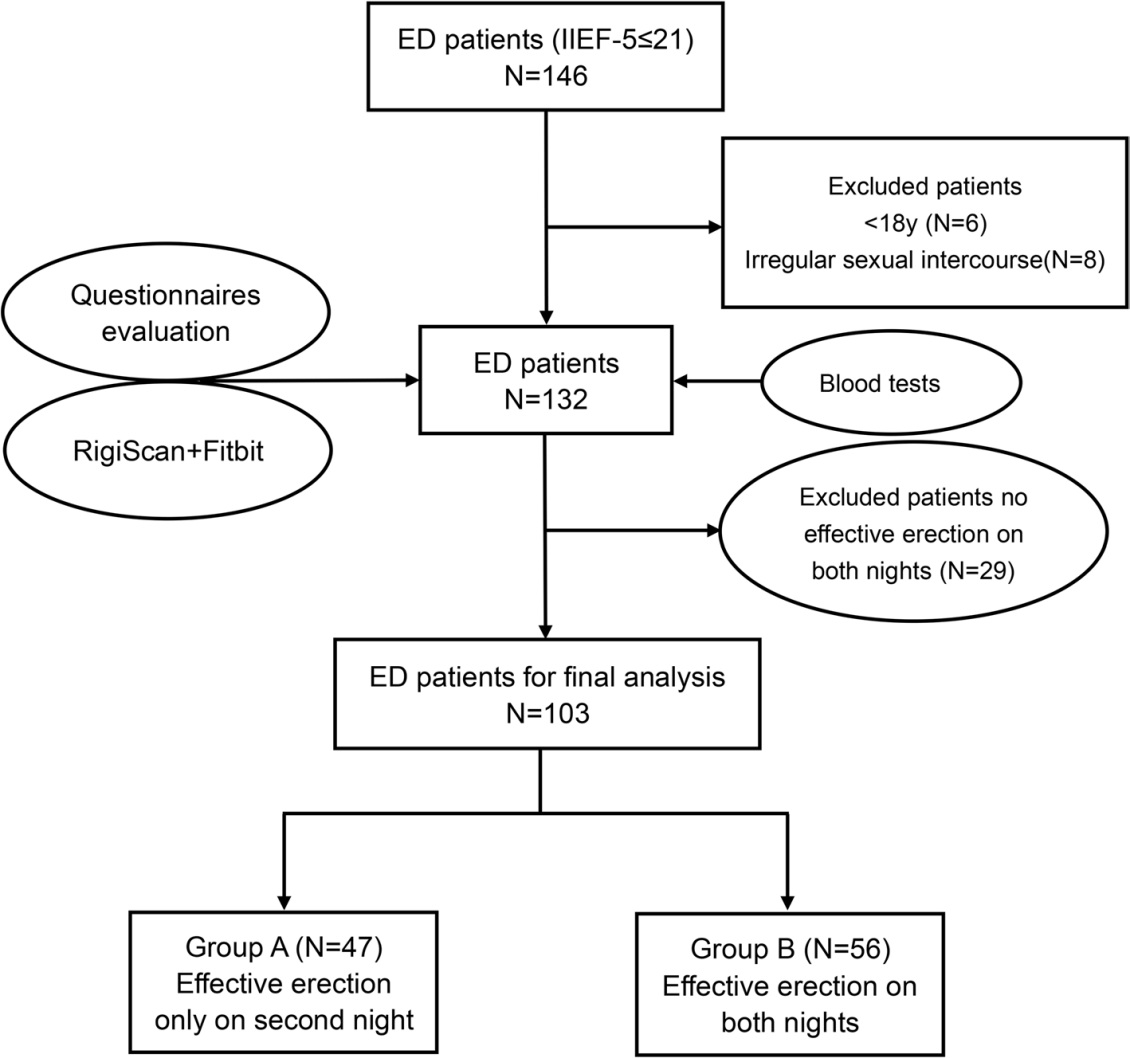
Flow diagram for patient selection process. ED: Erectile Dysfunction, IIEF-5: International Index of Erectile Function-5, RigiScan: device for detecting Nocturnal Penile Tumescence and Rigidity (NPTR), Fitbit: Fitbit Charge 2™ for sleep monitor; Group A and Group B were the two groups based on the NPTR results

Initially, a total of 146 consecutive ED patients were primarily screened for inclusion in our study. Among them, fourteen patients were excluded owing to their younger age (*n* = 6) or irregular sexual intercourse (*n* = 8). After completing the clinical questionnaires and blood tests, the remaining 132 patients underwent simultaneous nocturnal erection monitoring and sleep monitoring. Then, an additional 29 patients who did not exhibit any effective erections during either night were excluded from the analysis. Finally, a total of 103 patients aged between 18 and 40 years were included for analysis. The detailed process of patient selection was depicted in Fig. 1.

### Population characteristics of two group

Group A consisted of a total of 47 patients who did not exhibit any effective erection on the first night but had at least one effective erection on the second night. Group B included 56 patients who showed at least one effective erection on both nights. The mean age of Group A was 31.23 ± 6.58 years, while the mean age of Group B was 29.46 ± 10.38 years. There was no statistically significant difference in age between the two groups. Additionally, other baseline characteristics were compared between Group A and Group B, and no significant differences were observed. Detailed information can be found in Table 1.

**Table 1:** Clinical characteristics of study population

Parameters	Group A (*N* = 47)	Group B (*N* = 56)
Age, years	31.23 ± 6.58	29.46 ± 10.38
BMI, kg/m^2^	25.05 ± 3.60	24.43 ± 2.99
Personal history		
Smoking	22/47	35/56
Alcohol use	21/47	21/56
Regular exercise	24/47	27/56
Questionnaire evaluations		
IIEF-5	16.26 ± 3.29	17.21 ± 2.69
GAD-7 score	2.79 ± 2.01	2.68 ± 1.66
PHQ-9 score	3.66 ± 1.96	3.57 ± 1.73
ISI score	5.72 ± 4.03	4.71 ± 3.55
Blood tests		
FBG, mmol/L	4.75 ± 0.79	4.68 ± 0.70
TC, mmol/L	4.03 ± 0.68	4.14 + 0.90
TG, mmol/L	1.60 ± 0.74	1.76 ± 1.08
TT, nmol/L	16.38 ± 5.52	15.54 ± 5.27

*BMI* Body mass index, *IIEF-5* 5-question short version of the International Index of Erectile Function, *GAD-7* General Anxiety Disorder‐7, *PHQ-9* Patient Health Questionnaire‐9, *ISI* Insomnia Severity Index, *FBG* Fasting blood glucose, *TC* Total cholesterol, *TG* Triglycerides, *TT* Total testosterone (TT);

Group A and Group B were the two groups based on the NPTR results;

Data are presented as mean ± SD or as percentages (%);

Differences between Group A and Group B were assessed by independent t-test as appropriate

### Comparisons of NPTR and sleep parameters in two nights between Group A and Group B

In Group A, the total test time (TST) between the first and second nights did not show a significant difference (9.03 ± 0.30 h vs. 9.08 ± 0.21 h). However, all other NPTR parameters, including EEE, TET, Tip D60%, Base D60%, Tip AER, and Base AER, showed a significant improvement on the second night. Regarding the sleep parameters in Group A, total sleep time (TST), deep sleep, and REM sleep showed a significant improvement on the second night, while wake after sleep onset (WASO) decreased significantly. Please refer to Table 2 for detailed data.

**Table 2:** NPTR and Fitbit parameters of two consecutive nights in Group A and Group B

Parameters	Group A (*n* = 47)		Group B (*n* = 56)	
	N1	N2	N1	N2
NPTR				
TTT(h)	9.03 ± 0.30	9.08 ± 0.21	9.11 ± 0.27	9.12 ± 0.19
EEE(n)	0	2.21 ± 0.88*	2.38 ± 0.86	2.54 ± 1.11
TET(h)	0.57 ± 0.83	1.27 ± 0.76*	1.35 ± 0.45	1.38 ± 0.53
Tip D60%	8.49 ± 4.38	41.26 ± 18.97*	37.54 ± 14.95	47.59 ± 20.82*
Base D60%	12.43 ± 7.20	44.47 ± 19.66*	45.25 ± 15.47	50.66 ± 18.43*
Tip AER	37.77 ± 10.50	66.89 ± 13.91*	66.68 ± 15.90	68.75 ± 15.53
Baes AER	44.87 ± 14.26	67.85 ± 13.98*	69.50 ± 12.55	71.25 ± 12.71
Fitbit				
TST (min)	422.60 ± 51.05	472.55 ± 24.52*	474.71 ± 24.30	476.55 ± 30.91
SOL (min)	23.34 ± 9.59	22.40 ± 9.88	22.05 ± 10.67	21.02 ± 16.26
WASO (min)	98.72 ± 47.00	49.70 ± 16.15*	51.57 ± 13.68	49.80 ± 14.61
Time in N1 + N2 (min)	301.81 ± 63.93	292.17 ± 52.60	291.43 ± 48.16	292.73 ± 51.15
Time in N3 (min)	62.34 ± 17.46	72.13 ± 20.70*	73.16 ± 16.08	73.41 ± 22.80
Time in REM (time)	58.45 ± 30.57	108.26 ± 41.23*	110.13 ± 35.94	109.46 ± 34.78

*NPTR* Nocturnal penile tumescence and rigidity, *TTT* Total test time, *EEE* Effective erectile events, *TET* Total erection time, *D60%* Duration of erectile episodes with rigidity ≥ 60%, *AER* Average event rigidity, *TST* Total sleep time, *SOL* Sleep onset latency, *WASO* Wake after sleep onset, *REM* Rapid eye movement;

Group A and Group B were the two groups based on the NPTR results;

N1 and N2 were the first night and the second night for participants’ examination;

Data are presented as mean ± SD;

Differences between the first night and the second night within Group A and Group B were assessed by paired sample t tests;

^*^ *P* < 0.05 when measurement on the second night was compared with that on the first night

**Fig. 2:**
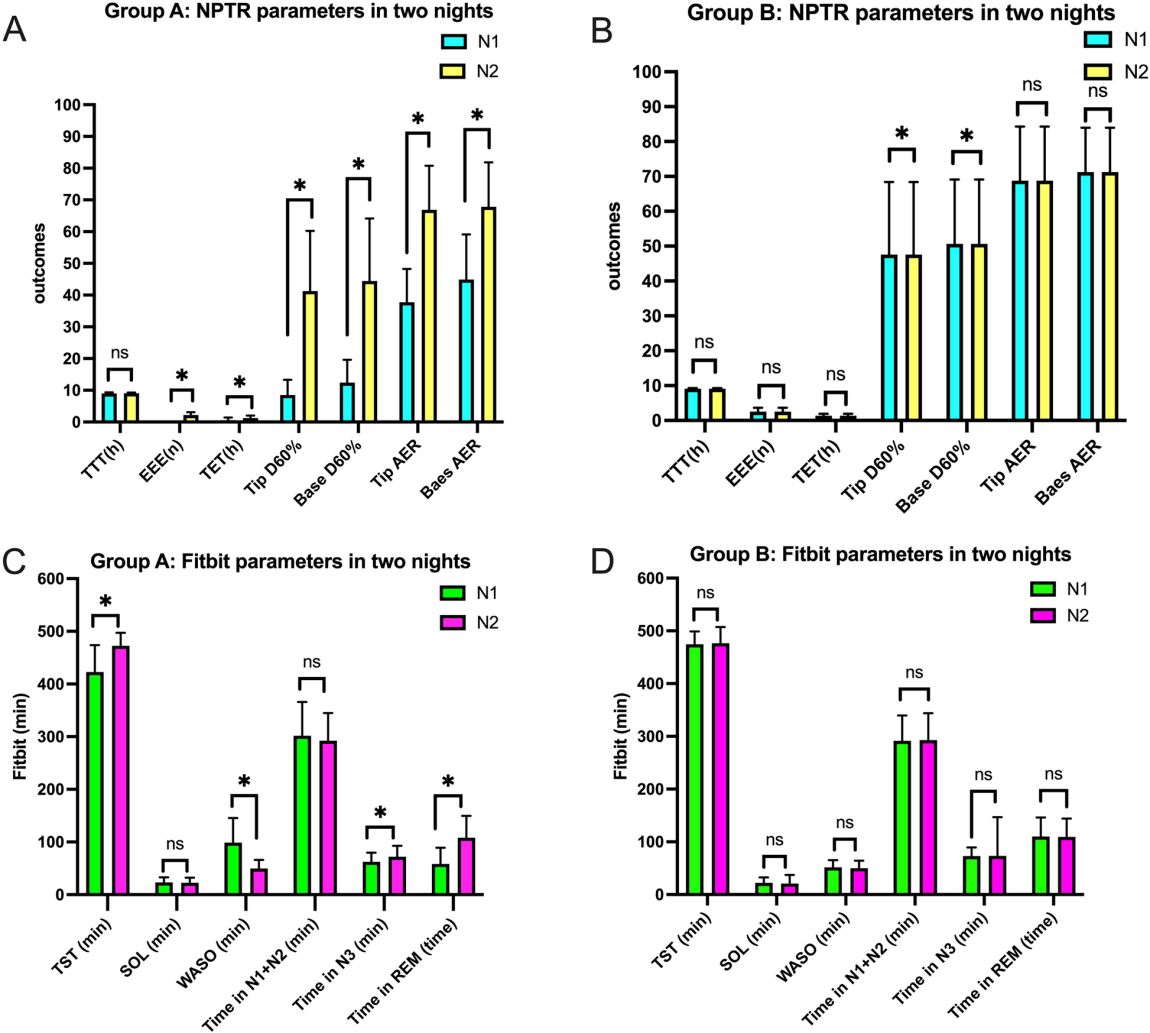
Comparisons of NPTR and Fitbit parameters in two nights between Group A and Group B. **A** NPTR parameters of Group A in two nights; **B** NPTR parameters of Group B in two nights; **C** Fitbit parameters of Group A in two nights; **D** Fitbit parameters of Group B in two nights. NPTR: Nocturnal Penile Tumescence and Rigidity; Fitbit: Fitbit Charge 2™; TTT: total test time; EEE: effective erectile events; TET: total erection time; D60%: duration of erectile episodes with rigidity ≥ 60%; AER: average event rigidity; TST: total sleep time; SOL: sleep onset latency; WASO: wake after sleep onset; REM: rapid eye movement; Group A and Group B were the two groups based on the NPTR results; N1 and N2 were the first night and the second night for participants’ examination; Differences between the first night and the second night within Group A and Group B were assessed by paired sample t tests; *: *P* < 0.05 when measurement on the second night was compared with that on the first night; ns: *P* > 0.05 when measurement on the second night was compared with that on the first night

In Group B, only Tip D60% and Base D60% showed a significant improvement on the second night. The remaining NPTR parameters did not show a significant improvement or decrease between the two nights. Additionally, none of the sleep parameters showed any changes between the two nights in Group B. Detailed data can be found in Table 2. For easier comprehension, the detailed comparisons are also presented in Fig. 2.

### Comparisons of NPTR and sleep parameters in two groups between first night and second night

Initially, the NPTR parameters on the first night were compared between Group A and Group B, revealing significant differences in all NPTR parameters except for TTT. However, when comparing the NPTR parameters on the second night between Group A and Group B, no significant differences were observed in any of the parameters.

**Fig. 3:**
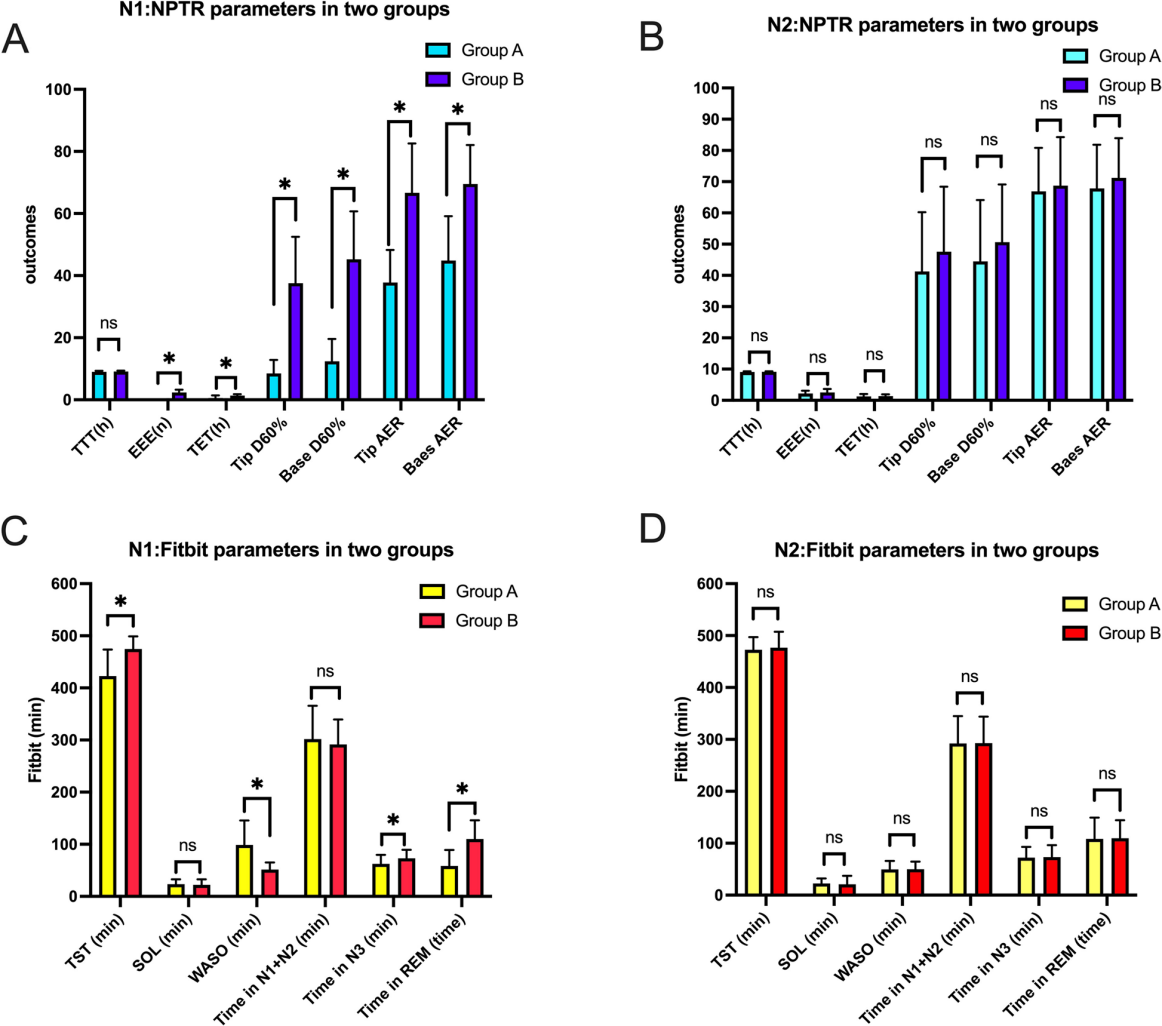
Comparisons of NPTR and Fitbit parameters in two groups between first night and second night. **A** NPTR parameters of the first night in Group A and Group B; **B** NPTR parameters of the second night in Group A and Group B; **C** Fitbit parameters of the first night in Group A and Group B; **D** Fitbit parameters of the second night in Group A and Group B. NPTR: nocturnal penile tumescence and rigidity; TTT: total test time; EEE: effective erectile events; TET: total erection time; D60%: duration of erectile episodes with rigidity ≥ 60%; AER: average event rigidity; TST: total sleep time; SOL: sleep onset latency; WASO: wake after sleep onset; REM: rapid eye movement; Group A and Group B were the two groups based on the NPTR results; N1 and N2 were the first night and the second night for participants’ examination; Differences between Group A and Group B within the first night and the second night were assessed by independent t tests; *: *P* < 0.05 when measurement on Group A was compared with that on Group B; ns: *P* > 0.05 when measurement on Group A was compared with that on Group B

Subsequently, the sleep parameters between the two groups were compared for both the first and second nights. On the first night, significant differences were found between the two groups in terms of total sleep time (TST), wake after sleep onset (WASO), deep sleep, and REM sleep duration. However, on the second night, similar to the NPTR parameters comparison, there were no significant differences in sleep parameters between the two groups. The detailed results can be found in Table 3 and Fig. 3.

**Table 3:** NPTR and Fitbit parameters in two groups between night one and night two

Parameters	N1		N2	
	Group A	Group B	Group A	Group B
NPTR				
TTT(h)	9.03 ± 0.30	9.11 ± 0.27	9.08 ± 0.21	9.12 ± 0.19
EEE(n)	0	2.38 ± 0.86*	2.21 ± 0.88	2.54 ± 1.11
TET(h)	0.57 ± 0.83	1.35 ± 0.45*	1.27 ± 0.76	1.38 ± 0.53
Tip D60%	8.49 ± 4.38	37.54 ± 14.95*	41.26 ± 18.97	47.59 ± 20.82
Base D60%	12.43 ± 7.20	45.25 ± 15.47*	44.47 ± 19.66	50.66 ± 18.43
Tip AER	37.77 ± 10.50	66.68 ± 15.90*	66.89 ± 13.91	68.75 ± 15.53
Baes AER	44.87 ± 14.26	69.50 ± 12.55*	67.85 ± 13.98	71.25 ± 12.71
Fitbit				
TST (min)	422.60 ± 51.05	474.71 ± 24.30*	472.55 ± 24.52	476.55 ± 30.91
SOL (min)	23.34 ± 9.59	22.05 ± 10.67	22.40 ± 9.88	21.02 ± 16.26
WASO (min)	98.72 ± 47.00	51.57 ± 13.68*	49.70 ± 16.15	49.80 ± 14.61
Time in N1 + N2 (min)	301.81 ± 63.93	291.43 ± 48.16	292.17 ± 52.60	292.73 ± 51.15
Time in N3 (min)	62.34 ± 17.46	73.16 ± 16.08*	72.13 ± 20.70	73.41 ± 22.80
Time in REM (time)	58.45 ± 30.57	110.13 ± 35.94*	108.26 ± 41.23	109.46 ± 34.78

*NPTR* Nocturnal penile tumescence and rigidity, *TTT* total test time, *EEE* Effective erectile events, *TET* Total erection time, *D60%* Duration of erectile episodes with rigidity ≥ 60%, *AER* Average event rigidity, *TST* Total sleep time, *SOL* Sleep onset latency, *WASO* Wake after sleep onset, *REM* Rapid eye movement

Group A and Group B were the two groups based on the NPTR results;

N1 and N2 were the first night and the second night for participants’ examination;

Data are presented as mean ± SD;

Differences between Group A and Group B within the first night and the second night were assessed by independent t tests;

^*^ *P* < 0.05 when measurement on Group A was compared with that on Group B

### Correlations between sleep parameters and NPTR parameters in Group A

The correlations between sleep parameters and NPTR parameters in Group A were evaluated, considering the significant improvements observed between the first night and second night. In the correlation analysis, the sleep parameters included wake after sleep onset (WASO) and REM sleep duration, while the NPTR parameters included Tip D60% and total erection time (TET). The analysis focused on the changes in these parameters between the two nights in Group A.

**Fig. 4:**
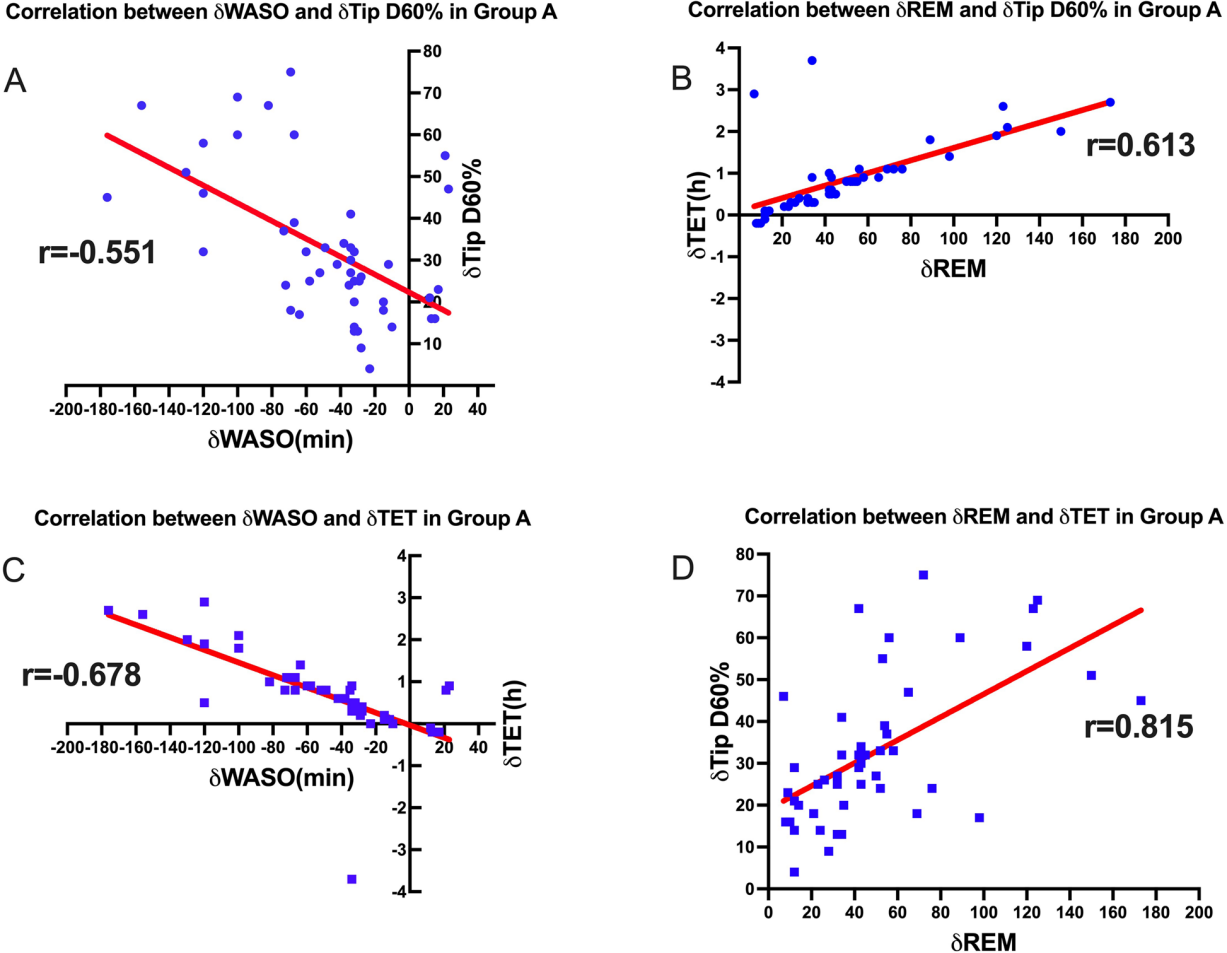
Correlations between changes of sleep parameters and changes of NPTR parameters in Group A. **A** Correlation between changes of WASO and changes of Tip D60% in Group A for two nights; **B** Correlation between changes of REM and changes of Tip D60% in Group A for two nights; **C** Correlation between changes of WASO and changes of TET in Group A for two nights; **D** Correlation between changes of REM and changes of TET in Group A for two nights. NPTR: nocturnal penile tumescence and rigidity; TET: total erection time; D60%: duration of erectile episodes with rigidity ≥ 60%; WASO: wake after sleep onset; REM: rapid eye movement; Group A was the group included participants presenting effective erectile events only on the second night; Correlation analyses were evaluated by Spearman’s and Pearson’s method as appropriate; *: *P* < 0.05 for the correlation analysis; ns: *P* > 0.05 for the correlation analysis

The results revealed that an increase in WASO time was negatively correlated with the increase in Tip D60% and TET. On the other hand, an increase in REM sleep duration was positively correlated with the increase in Tip D60% and TET. These correlations suggest that a longer duration of wakefulness after sleep onset (WASO) was associated with a decrease in the rigidity of nocturnal erections (Tip D60%) and TET. Conversely, a longer duration of REM sleep was linked to an increase in the rigidity of nocturnal erections and total erection time. Please refer to Table 4 and Fig. 4 for detailed results.

**Table 4:** Correlations between NPTR parameters changes and Fitbit parameters changes in Group A

		NPTR	
	Parameters	Tip D60%	TET
Fitbit	WASO	-0.551*	-0.678*
	REM	0.613*	0.815*

*NPTR* Nocturnal penile tumescence and rigidity, *TET* Total erection time, *D60%* Duration of erectile episodes with rigidity ≥ 60%, *WASO* Wake after sleep onset, *REM* Rapid eye movement;

Group A was the group included participants presenting effective erectile events only on the second night;

Correlation analyses were evaluated by Spearman’s and Pearson’s method as appropriate;

^*^ *P* < 0.05 for the correlation analysis

### Predictive effects of sleep parameters on abnormal NPTR results

Following the correlation analysis, we conducted ROC curve analysis to assess the predictive value of the sleep parameters, specifically WASO and REM sleep, for abnormal NPTR results.

For the WASO time, the cut-off value of 67.5 min was identified to predict abnormal NPTR results, with an area under the curve (AUC) of 0.863 (95% confidence interval (CI): 0.785–0.941). In other words, if the patients' WASO time during sleep exceeded 67.5 min, the NPTR results were more likely to be unreliable.

**Fig. 5:**
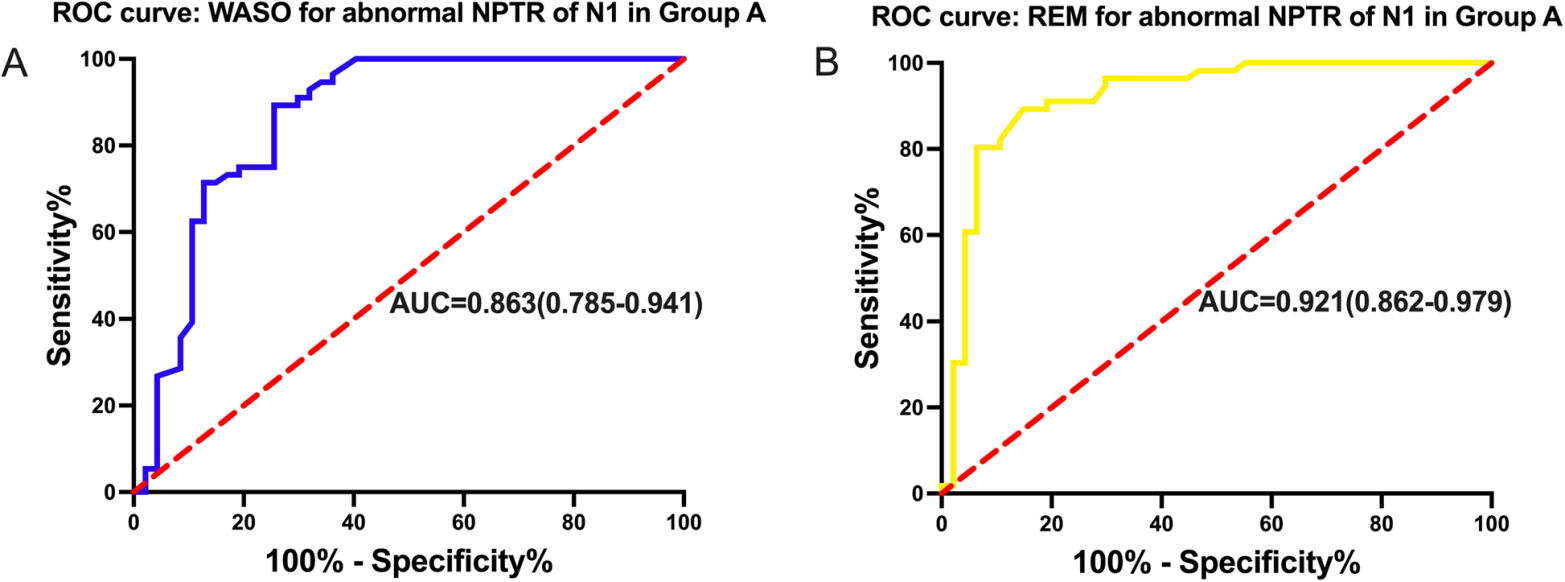
Receiver operator characteristic curves of sleep parameters for predicting abnormal NPTR in Group A. **A** ROC curve of WASO for abnormal NPTR test of N1 in Group A, **B** ROC curve of REM for abnormal NPTR test of N1 in Group A. ROC: Receiver Operator Characteristic curves, REM: Rapid Eye Movement, WASO: Wake After Sleep Onset, AUC: Area Under Curve, N1: the first night; Group A: included participants presenting effective erectile events only on the second night

Similarly, the cut-off value of REM sleep duration was determined to be 77 min for predicting abnormal NPTR results, with an AUC of 0.921 (95% CI: 0.862–0.979). If the patients' REM sleep time during sleep was shorter than 77 min, the NPTR results were more likely to be unreliable. Please refer to Table 5 and Fig. 5 for detailed results.

**Table 5:** Receiver operator characteristic curves for sleep parameters to predict abnormal NPTR results

Parameters	Cut-off values	AUC	Sensitivity	Specificity	*P* values
WASO, min	67.5	0.863	89.3%	74.5%	< 0.001
REM, min	77	0.921	89.3%	85.1%	< 0.001

*NPTR* Nocturnal penile tumescence and rigidity, *WASO* Wake after sleep onset, *REM* Rapid eye movement, *AUC* Area under curve

## Discussion

Based on the previous research conducted in our research group [24, 35], the present study furtherly confirmed the feasibility of using Fitbit for monitoring sleep in patients with ED. Our results revealed that the results of NPTR measurements were negatively influenced by sleep quality, particularly REM sleep time. The combination of RigiScan with Fitbit monitoring proved to be more valuable compared to using RigiScan alone. This combined approach provides physicians with more reliable results for distinguishing between different etiologies of ED. It was observed that only patients with normal sleep quality had NPTR results that accurately reflected their erectile function.

Among 132 patients of our study finishing the erection and sleep monitor, 56 patients (42.4%) had at least one effective erection on the first night. According to the guidelines on erectile dysfunction [36], a functional erectile mechanism is indicated in them. Their clinical diagnoses were not be changed based on the second night measurement, although, significant improvement of some NPTR parameters. When comparing their sleep parameters between two nights, no significant improvements were observed. In other words, they were not influenced by the “first-night effect”, and their first-night measurement could accurately reflect their reliable erectile function based on normal sleep. In a previous study conducted by Zou et al., sixty-five patients of 105 patients (61.9%) showed at least one effective erectile event on both nights [13]. However, it is important to note that in their study, simultaneous sleep monitoring was not performed. This suggests that the “first-night effect” may not occur on every patient undergoing NPTR test. Therefore, there may not be a necessity to conduct consecutive nightly measurements for every patient.

However, it is undeniable the NPTR measurement of some patients on the first night would be affected by the “first-night effect” [36, 37]. The occurrence of SREs were closely associated with the REM sleep [38], although the exact mechanism was not elucidated. In our study, the positive changes of NPTR parameters between the first and second night were related tightly with the positive changes of REM sleep time. In another study, the nocturnal erection monitors were performed combining with PSG, and their results also supported our conclusion [37]. Through our ROC analysis, the REM sleep time owned the highest area under the curve, the best parameter to predict abnormal NPTR results. Therefore, we concluded that the adequate duration of REM sleep time is the basis of SREs, and if the REM sleep time decreased seriously, it compromises the foundation for the occurrence of SREs.

Contrary to our study, a prior study, owing the similar study design with us, demonstrated that multiple NPTR parameters were significant better on the first time than those on the second time [39]. Nevertheless, the total test time between two nights showed significant difference, that a longer total test time on the first night than that on the second night. Although no objective sleep monitors were conducted on their study, we speculated that the extended test time on the first night improve the sleep efficacy, and improved sleep efficacy shrunk the impact of sleep on the nocturnal erection. Consequently, it is absolutely necessary to objectively monitor sleep simultaneously, when performing nocturnal erection monitor, therefore avoiding the misdiagnosis. When interpreting the NPTR results, the result was reliable if the corresponding sleep parameters were normal. On the contrary, if the corresponding sleep parameters were abnormal, the reliability of the result became questionable. For those with normal sleep parameters, repeated NPTR measurements were unnecessary, as their initial measurement could provide reliable results.

A previous study suggested that the main reason for patients experiencing the first-night effect may be the anxiety they feel in the hospital testing environment [40]. Naturally, it raises the question of whether conducting the NPTR tests at home could alleviate this anxiety and improve the accuracy of the examinations. However, current research remains skeptical about home-based testing. One study indicated that patients undergoing home-based NPTR tests had more interruptions and shorter testing durations, suggesting that home-based NPTR tests does not completely eliminate the first-night effect [41]. Therefore, further research is needed in the future to explore the factors that may influence patients' test results, aiming to enhance the accuracy and efficiency of the examinations.

From the perspective of clinical diagnosis, the eligible ED patients of our study could be diagnosed with psychogenic ED, based on their normal NPTR parameters. Other 29 patients, excluded from our analysis, may be diagnosed with organic ED, based on their abnormal NPTR parameters on both nights. Several impairments in organic ED, including penile tissues, vessels and nerves [42], could negatively affect the quality of nocturnal erection. For these organic ED patients, the NPTR parameters might reflect the reliable erectile function, only when their sleep monitors were normal. On the prior study, they found that the NPTR parameters on the second night didn’t improve significantly, when comparing to the first night [13]. They speculated that the extend of negative impact of the “first night effect” was limited owing to the impaired erectile function by organic diseases. However, no related study was conducted to verify their opinions, considering the complex relationship between ED and sleep disorder.

Actually, the SREs occurring during REM sleep could supply cavernous tissues with periodic oxygenation, which ensured adequate activity of nitric oxide synthase to maintain the erectile function [43]. So, more attention has been increasingly paid to the association between ED and sleep disorder, including obstructive sleep apnea, Insomnia, and chronic sleep insufficiency [44]. An interesting study conducted by Lee et al. [45] to assess the association between nocturnal frequency and erectile dysfunction in patients with benign prostatic obstruction. They found that the sleep fragmentation due to nocturnal frequency could interfere with the amount and quality of SREs, therefore producing negative impact on the erectile function. But only the international prostate symptom score questionnaire was used in study, instead of objective monitor of sleep quality. Recently, another study was conducted by our group to evaluate the association between sleep quality and ED [24]. Not only the subject Pittsburgh Sleep Quality Index questionnaires, but also the objective sleep monitor devices were used to indicate that poor sleep quality may be a risk factor for ED. Considering the above studies, the sleep monitor and evaluation allows both the exploration of possible risk factor and offering the reference for NPTR test. For ED patients with sleep disorders, especially sleep insufficiency, the abnormal NPTR results should be interpreted cautiously, explained either by the influence of sleep insufficiency, or by the organic impairment of erectile function.

During our form published article validating association between REM sleep and SREs, we found that nearly 90% erectile events were related to REM sleep. Additionally, the time of total erectile events was statistically related to the duration of REM sleep [13]. This study serves as the foundation for our research, but it differs from our current study. In that study, they included the entire duration of nocturnal erections, while we primarily focused on calculating the time with an erection rigidity exceeding 60%. Additionally, due to the inclusion of healthy individuals, their monitoring period was relatively short and did not reach 8 h. These differences in study design and participant characteristics contribute to variations in sleep and erection parameters between our two studies. Their study primarily aimed to further validate the relationship between SREs and REM sleep using Fitbit devices. In contrast, our study builds upon their findings and explores the clinical significance of combined monitoring, aiming to reduce unnecessary testing for patients.

Another application of our study was used to resolve legal disputes. The sleep monitor via PSG was advised by Herskowitz et al. during forensic cases, to prevent crimes from deliberately controlling sleep, therefore negatively impact the NPTR results [46]. But the PSG itself would negatively affect sleep quality, and using it would not still avoid disputes. From our study, the Fitbit device was verified for his role of sleep monitor in the NPTR tests, and it would improve the reliability of it in the legal cases, as well as clinical cases.

There are several limitations that should be acknowledged when interpreting our study conclusions. Firstly, the study sample size was relatively small, which may limit the generalizability of our results. Second, Secondly, it is important to note that our study only included patients with normal erectile function, which introduces a potential bias in our conclusions. Sleep disorders have been established as risk factors for organic erectile dysfunction (ED). However, it remains challenging to determine whether the observed abnormal sleep parameters were the underlying cause of ED or simply a result of the "first-night effect" commonly observed in sleep studies. Third, it is worth noting that in our study, we did not conduct a separate sleep monitoring assessment on the patients prior to the formal nocturnal erection monitoring. This aspect of sleep monitoring is an important consideration for future research. In our future studies, we plan to perform sleep monitoring on erectile dysfunction (ED) patients before conducting the formal nocturnal erection monitoring. This will allow us to obtain a baseline assessment of their sleep quality. The formal NPTR results and the sleep monitoring data will then be analyzed in relation to the referenced sleep quality obtained during the initial assessment. Lastly, the use of Fitbit Charge 2TM as a sleep monitoring device, while convenient and practical, may have certain limitations in terms of accuracy and reliability compared to gold standard methods of PSG.

## Conclusion

Based on our study findings, we can conclude that there is a strong association between sleep parameters, particularly REM sleep and wake after sleep onset (WASO), and NPTR parameters. When an individual with erectile dysfunction (ED) exhibits abnormal sleep parameters as monitored by the Fitbit Charge 2™, the reliability of their NPTR test results becomes questionable, warranting consecutive measurements. On the other hand, if the sleep parameters are within normal ranges, the NPTR results can be considered reflective of their objective erectile function, eliminating the need for repeated measurements. However, it is important to note that further well-designed studies with larger sample sizes and inclusion of various types of ED patients are required to validate our conclusions.

## Data Availability

The data used for the present study are available from the corresponding author on reasonable request.

## Abbreviations

AER: Average Event Rigidity
AUA: American Urological Association
CI: Confidence Interval
D60%: Duration of Erectile Episodes with Rigidity ≥ 60%
EAU: European Association of Urology
ED: Erectile Dysfunction
EEE: Effective Erectile Events
EEG: Electroencephalographic
EMG: Electromyographic
EOG: Electrooculographic
FBG: Fasting Blood Glucose
GAD-7: General Anxiety Disorder-7
IIEF-5: International Index of Erectile Function-5
ISI: Insomnia Severity Index
NPTR: Nocturnal Penile Tumescence and Rigidity
NPT: Nocturnal Penile Tumescence
PSG: Polysomnography
REM: Rapid Eye Movement
ROC: Receiver Operating Characteristic
SD: Standard Deviation
SOL: Sleep Onset Latency
SREs: Sleep-Related Erections
TET: Total Erection Time
TG: Triglycerides
Tip: Penile Tip
TST: Total Sleep Time
TT: Total Testosterone
TTT: Total Test Time
USA: United States of America
WASO: Wake After Sleep Onset
